# Withholding or continuing beta-blocker treatment before dipyridamole myocardial perfusion imaging for the diagnosis of coronary artery disease? A randomized clinical trial

**DOI:** 10.1186/2008-2231-21-8

**Published:** 2013-01-15

**Authors:** Babak Fallahi, Davood Beiki, Saeed Akbarpour, Ali Gholamrezanezhad, Armaghan Fard-Esfahani, Fariba Akhzari, Sina Izadyar, Javad Esmaeli, Mohsen Saghari, Mohammad Eftekhari

**Affiliations:** 1 Research Center for Nuclear Medicine, Tehran University of Medical Sciences, Tehran, Iran; 2 Department of Nuclear Pharmacy, School of Pharmacy, Tehran University of Medical Sciences, Tehran, Iran; 3 Department of Nuclear Medicine, Sina Hospital, School of Medicine, Tehran University of Medical Sciences, Tehran, Iran; 4 Department of Nuclear Medicine, Vali-Asr Hospital, School of Medicine, Tehran University of Medical Sciences, Tehran, Iran

**Keywords:** ^99m^Tc-MIBI, Beta-blocker, Dipyridamole, Myocardial perfusion imaging, Coronary artery disease

## Abstract

**Background:**

Although it has been shown that acute beta-blocker administration may reduce the presence or severity of myocardial perfusion defects with dipyridamole stress, little information is available about the potential effect of chronic beta-blocker treatment on the sensitivity of dipyridamole myocardial perfusion imaging (DMPI).

**Methods:**

As a randomized clinical trial, one hundred twenty patients (103 male and 17 female) with angiographically confirmed CAD who were on long-term beta blocker therapy (≥3 months) enrolled in a randomized clinical trial study. The patients were allocated into two groups: Group A (n=60) in whom the beta-blocker agent was discontinued for 72h before DMPI and Group B (n=60) without discontinuation of beta-blockers prior to DMPI.

****Results**:**

No significant difference was noted between the groups concerning age, sex, type of the injected radiotracer and number of involved coronary vessels. The mean rank of total perfusion scores for whole myocardium (irrespective of reversibility or irreversibility) in group B was not significantly different from that of group A, (65.75 vs. 55.25, P=0.096). Regarding the only irreversible perfusion defects, the mean rank of perfusion score in group B was higher than that of group A for whole myocardium (72 vs. 49, P=0.0001); however, no difference was noted between two groups for only reversible perfusion defects (61.0 vs. 60.0, P=0.898). The overall sensitivity of DMPI for the diagnosis of CAD in group A (91.7%) was not statistically different from group B (90%).

**Conclusion:**

Beta-blocker withholding before DMPI did not generally affect the sensitivity of the test for the diagnostic purposes in our study. Thus, beta-blocker withdrawal for just the purpose of diagnostic imaging is not mandatory particularly when medication discontinuation may cause the patients to face increased risk of heart events.

## Introduction

Scintigraphic myocardial perfusion imaging has been established as one of the most frequently used diagnostic tools in noninvasive assessment of the likelihood of coronary artery disease (CAD) [1-3]. Infusion of pharmacological vasodilators, including dipyridamole and adenosine or exercise treadmill test (ETT) are the main protocols of cardiac stressing in these imaging interventions, although less frequently other methods are also applied [4-6].

When ETT is selected as the stress method, discontinuation of agents affecting heart rate, most importantly beta-blockers and calcium channel blockers are usually advised, the underlying explanation of which is to allow heart rate to reach the age-predicted value [7]. However, there is some debate on the necessity of discontinuation of these agents for those patients undergoing pharmacological stress.

Some previous studies suggest that acute beta-blocker administration may reduce the presence and severity of myocardial perfusion defects with dipyridamole stress [8-17]. Nevertheless, most of such studies have been performed with short-term or acute beta-blocker treatment, some after intravenous administration, rather than after long-term oral beta-blocker treatment so that their methods differ from the usual clinical scenario encountered in many patients referring for dipyridamole myocardial perfusion imaging (DMPI) [8-17]. Few studies suggested that coronary flow reserve measured by means of positron emission tomography (PET) is improved in stenosis-dependent segments of the myocardium during long-term beta-blocker treatment, thereby b-blockers may decrease the contrast between ischemic and non-ischemic myocardium during hyperemia induced by dipyridamole [18]. However, they used metoprolol as a selective beta-1 receptor blocker and carvedilol as a non-selective beta blocker/alpha-1 blocker and thus this effect may not be generalized to other nonselective beta-blockers such as propranolol. To our knowledge, no clinical trial has been performed so far on the basis of DMPI with single-photon emission tomography (SPET) with random continuation or discontinuation of long-term beta-blocker medication, to study the effect of discontinuing chronic beta-blockade on the sensitivity of DMPI.

The current study was designed to evaluate the effect of discontinuing vs. continuing beta-blocker drugs on DMPI in patients who were on long-term treatment with these drugs.

## Methods

### Study population

The study was approved by the committee on ethics of Tehran University of Medical Sciences. All patients gave written informed consent before entering the study.

One hundred twenty patients (103 male and 17 female) with angiographically confirmed CAD (i.e. more than 50% diameter stenosis in at least one coronary artery or major branches), who were on long-term treatment (≥3 months) with therapeutic dose of a beta blocker, enrolled in a randomized clinical trial. Patients with a past history of asthma, second degree type 2 or third degree atrio-ventricualr block, left ventricular ejection fraction less than 50%, previous angioplasty and/or coronary artery bypass graft were excluded from the study.

The patients were allocated into two groups, using permuted block randomization method [19]: Group A (n=60) in whom the beta-blocker agent was discontinued for 72 h before DMPI (i.e. for more than 5 drug half-lives for all beta-blockers used in the study) and Group B (n=60) without discontinuation of beta-blockers prior to DMPI. After randomization of patients, the average number of stenosed arteries in group A was 2.0 and in group B was 2.2 with no significant difference between groups (P=0.180). No patient with left main coronary artery disease was included in each group of the study. The similarity of the two groups related to the age, gender, dose of beta-blockers before randomization, severity, extent and significance of CAD is shown in Table 1.

**Table 1:** Comparison of baseline variables between group of patients with discontinued beta-blocker (Group A) and the group on beta-blockade (Group B)

		**Group A ** **N=60**	**Group B ** **N=60**	**P Value**
**Age (years)**		55.63±12.23	52.18±10.16	0.096 (NS)*
**Gender**	Male	55 (91.7%)	48 (80%)	0.114 (NS)
	Female	5 (8.3%)	12 (20%)	
**Radiotracer**	^201^Tl	40 (66.70%)	42 (70%)	0.845 (NS)
	^99m^Tc-MIBI	20 (33.30%)	18 (30%)	
**Average dose of beta-blocker (mg/day)**	Propranolol	53.2±22.4	45.7±20.1	0.230 (NS)
	Metoprolol	68.5±17.3	61.7±11.7	0.375 (NS)
	Atenolol	46±10.8	50.0±6.3	0.464 (NS)
**Number of involved coronaries**	One	20 (33.30%)	12 (37.5%)	0.256 (NS)
	Two	20 (33.30%)	24 (54.5%)	
	Three	20 (33.50%)	24 (54.5%)	
Significant LAD** **stenosis**		46 (81.8%)	53 (88.3%)	0.093 (NS)

*NS: Non-significant.

**LAD: left anterior descending artery.

(No significant difference was noted between the groups concerning age, sex, type of the radiotracer injected, average dose of beta-blockers being administered orally before randomization, number of involved coronary vessels and the frequency of LAD stenosis).

### SPET imaging

Patients were instructed to fast for at least 4h before stress phase imaging. Caffeine, theophylline or aminophylline were discontinued for 48 h before the pharmacological stress. For stress-phase imaging, a dose of 0.56 mg/kg dipyridamole was infused intravenously (i.v.) over a 4 min period. Radiotracer was injected i.v., 3-5 min after the completion of dipyridamole infusion. Post-stress imaging was performed following the injection of 925MBq technetium-99m methoxyisobutyl isonitrile (^99m^Tc-MIBI) or 111MBq thallium-201 (^201^Tl) after dipyridamole infusion. Half an hour after injection of ^99m^Tc-MIBI, the patients were encouraged to eat a fat-rich snack to accelerate hepatobiliary excretion of the radiotracer. Cardiac SPET acquisition was performed 0.5 h after the fatty meal or one hour after i.v. injection of the radiopharmaceutical. For ^201^Tl SPET, image acquisition was started 10-15 min after the radiotracer injection.

In the case of imaging with ^99m^Tc-MIBI, the rest phase of the study was performed in the following day using injection of the same dose of ^99m^Tc-MIBI at rest, while for ^201^Tl SPET myocardial perfusion imaging, it was done in the same day using re-injection of 37MBq ^201^Tl.

For ^99m^Tc-MIBI SPET, image acquisitions were done using a rotating, dual head gamma camera (Solus, ADAC, Milpitas, CA) equipped with a low-energy high-resolution parallel-hole collimator. Patients were in a supine position during the SPET acquisition. Thirty-two azimuth images, 60s/projection, were obtained in a 180° circular orbit, beginning from 45° right anterior oblique to 135° left posterior oblique with step and shoot acquisition on a 128×128×16 matrix and 38.5 cm detector mask. For ^201^Tl SPET, all imaging parameters were similar except for using low-energy all-purpose collimator and 64×64×16 matrix. Filtered back-projected data was reconstructed into short-axis, vertical long-axis and horizontal long-axis slices.

**Figure 1:**
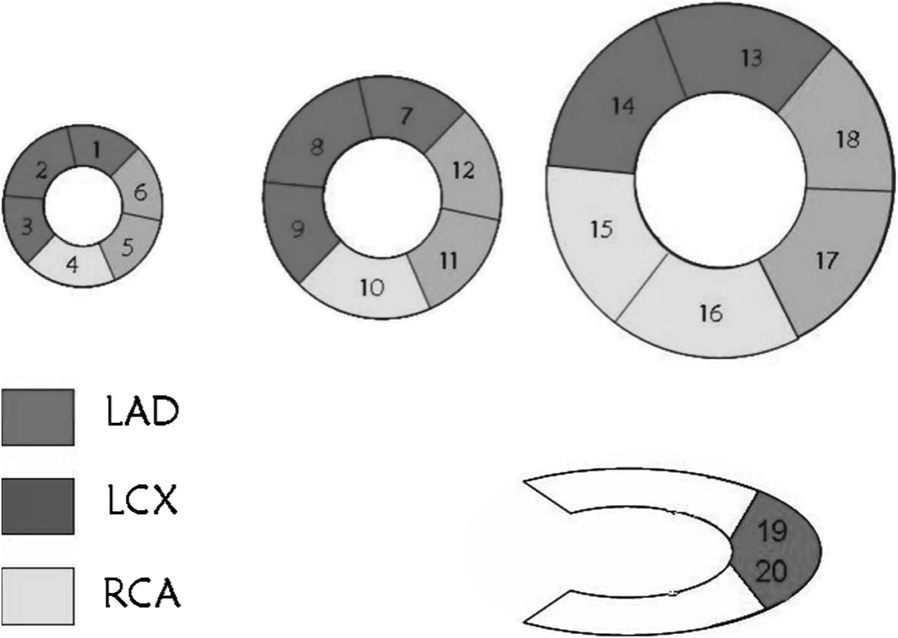
**The segmentation pattern of myocardium to define different coronary territories.** (The entire left ventricular myocardium was divided into 20 segments according to a predetermined 20-segment model).

Images were interpreted by three expert nuclear medicine physicians blinded to the patients’ clinical data and final decision was reached by consensus. For this purpose, the entire left ventricular myocardium was divided into 20 segments according to a predetermined 20-segment model (Figure 1). At first, the images were qualitatively interpreted as positive or negative for the presence of any perfusion defect in more than one segment throughout the myocardium. Thereafter, visual semiquantitative analysis was performed considering a variety of artefactual findings that can be identified only by visual assessment. The perfusion status in each segment was classified as normal, reversible or irreversible defect by visual comparison of the intensity of myocardial color-coded scintillation on the stress and rest images. The total reversible and irreversible perfusion scores for each patient were calculated separately based on the number of segments with any degree of reversible or irreversible perfusion defects, respectively.

### Statistical analysis

Considering the angiographic results as the gold standard, the sensitivity of DMPI for the detection of CAD in each group was calculated based on 95% confidence interval (95%CI). The combined data from the perfusion scores of all patients in both groups were sorted and ranked from 1, assigned to the lowest score, to 120 intended to the highest score. The average rank in group A was compared to group B using Mann–Whitney-U-test. In this way, the number of times that a score from group A precedes a score from group B and the number of times that a score from group B precedes a score from group A were analyzed. SPSS for Windows (Release 11.5.0) was used for statistical analysis. The significance level was 0.05.

## Results

No significant difference was noted between the groups concerning age, sex, type of the radiotracer injected, average dose of beta-blockers being administered orally before randomization, number of involved coronary vessels and the frequency of left anterior descending artery (LAD) stenosis (Table 1).

**Figure 2:**
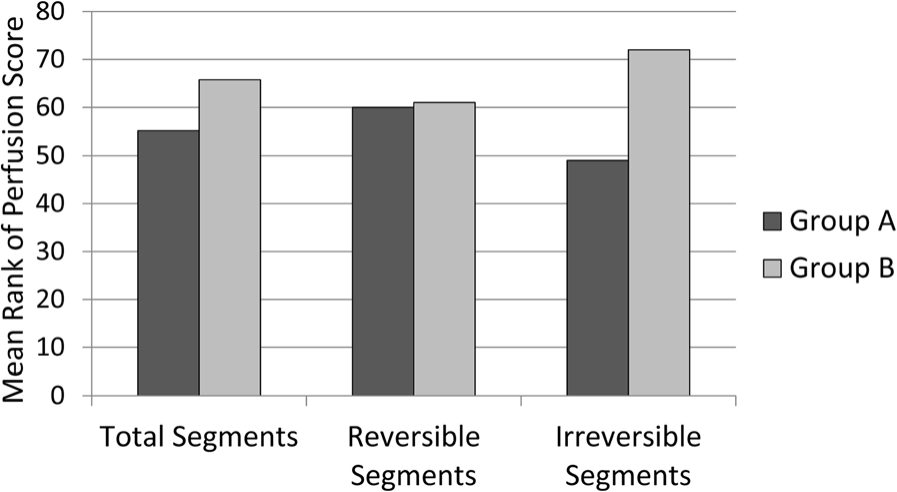
**Comparison of the mean rank of perfusion score for total, reversible and irreversible segments between patients discontinued beta-blocker (Group A) and those on beta-blockade (Group B) during dipyridamole myocardial perfusion imaging.** (The mean rank of total perfusion scores for whole myocardium, irrespective of reversibility or irreversibility, in group B (65.75) was not significantly different from that of group A (55.25, P=0.096). Regarding the only irreversible perfusion defects, the mean rank of perfusion score in group B was substantially higher than that of group A for whole myocardium (72 vs. 49, P=0.0001); however, no difference was noted between group A and B for only reversible perfusion defects (60.0 vs. 61.0, respectively, P=0.898)).

The mean rank of total perfusion scores for whole myocardium, irrespective of reversibility or irreversibility, in group B (65.75) was not significantly different from that of group A (55.25, P=0.096) (Figure 2). Regarding the only irreversible perfusion defects, the mean rank of perfusion score in group B was substantially higher than that of group A for whole myocardium (72 vs. 49, P=0.0001), (Figure 2); however, no difference was noted between group A and B for only reversible perfusion defects (60.0 vs. 61.0, respectively, P=0.898).

The overall sensitivity of DMPI for the diagnosis of CAD in group A was 91.7% (95%CI: 83.1%-98.7%) and in group B was 90% (95%CI: 82.2%-97.8%). The difference was not statistically significant (P=0.945).

Additional analysis was performed based on the type of beta-blockers. For this purpose, the patients in groups A and B were divided into two subgroups. The first subgroup included the patients who were taking selective β1 receptor antagonists (i.e. metoprolol and atenolol) and the second subgroup was composed of those who were on nonselective β1/β2 receptor antagonists (i.e. propranolol and carvedilol) before entering the study. The sensitivity of DMPI for the diagnosis of CAD did not differ between groups, A and B, even across separate subgroups of patients based on the type of beta-blocker, gender and age (Table 2).

**Table 2:** Sensitivity of dipyridamole myocardial perfusion imaging (DMPI) in two groups, based on age, gender and type of beta-blocker agent

		**Group A**	**Group B**	**P value**
**Age**	≤45 Years	(19/19) 100.0%	(18/18) 100.0%	1.00
	>45 Years	(36/41) 87.8%	(36/42) 85.7%	0.940
**Gender**	Male	(50/55) 90.9%	(42/48) 87.5%	0.894
	Female	(5/5) 100.0%	(12/12) 100.0%	1.00
**Type of beta-blocker**	Nonselective β1/β2 antagonist	(35/35) 100.0%	(42/48) 87.5%	0.675
	Selective β1antagonist	(20/25) 80.0%	(12/12) 100.0%	0.659
	Propranolol	(35/35) 100.0%	(36/42) 85.7%	0.640
**Beta-blocker**	Carvedilol	(0/0)	(6/6) 100.0%	-
	Atenolol	(5/5) 100.0%	(6/6) 100.0%	1.00
	Metoprolol	(15/20) 75%	(6/6) 100.0%	0.667

Group A: patients with discontinued beta-blockade; Group B: patients on beta-blockade.

(The sensitivity of DMPI for the diagnosis of coronary artery disease did not differ between groups, A and B, even across separate subgroups of patients based on the type of beta-blocker, gender and age).

## Discussion

Based on our study findings, the diagnostic sensitivity of DMPI is not significantly affected by the chronic consumption of beta-blockers. There is a common and overlapping sub-cellular and molecular pathways of action between dipyridamole and beta-blockers. Dipyridamole increases the endogenous concentration of adenosine, the underlying mechanism of which is inhibition of adenosine deaminase (ADA) enzyme. Adenosine in turn binds to A2 receptors and subsequently enhances the activity of its corresponding G-proteins. The cascade will eventually lead to increase in cellular concentration of cAMP, an event which leads to vasodilatation [20-24]. In atherosclerotic coronaries, vasodilatory response is restricted leading to the shift of blood to normal coronaries (steal phenomenon). This event is the rationale for using dipyridamole infusion to unveil possible myocardial ischemia [25-30]. On the other hand, beta adrenergic receptors bind to the same stimulatory G-proteins [31,32]. Therefore, theoretically it can be judged that inhibitors of such receptors may decrease activation of the corresponding G-proteins, which eventually lead to decreased cellular concentration of cAMP and reduced response to dipyridamole infusion [17,26,33]. Based on these facts, others concluded that “much of the coronary vasodilation associated with hypoxia is dependent on adrenergic activation and that adenosine may only play a role in sustained hypoxic vasodilation when adrenergic receptors are intact” [33]. Such a probable effect practically could result in reduced sensitivity of DMPI. This unpleasant outcome was supposed to be easily avoided with discontinuation of beta-blockers before stress protocol of dipyridamole infusion.

On the other hand, dipyridamole infusion causes an increase in rate-pressure product, which can be considered as one of the main explanations for dipyridamole-induced steal phenomenon. The masking effect of beta-blockade on such an index has been considered as another reason for decreased sensitivity of DMPI [34].

These hypotheses were supported with findings of some reports, which showed decreased sensitivity of DMPI in those patients with history of calcium channel blocker or beta-blocker treatment [34-39], although most of them were retrospective and suffered from limitations such as low sample size, lack of gold standard, lack of individual and separate evaluation of beta-blocker effect rather than combined effect of multiple anti-anginal drugs [36] and acute versus chronic beta blockade [9]. Furthermore, controversies exist so far, as our study and also some of the other reports demonstrate opposite findings [14,40-42].

According to the previous assumptions, beta-blocker withdrawal in patients with long-term beta-blockade is expected to result in restoring adrenergic activation and consequently better dipyridamole effect [33], rising the heterogeneity of regional perfusion between stress and rest phases, leading to better detection of reversible defects that might have been overlooked in case of ongoing beta-blocker treatment. Conversely, in our study, enduring beta-blocker treatment in the time of DMPI was associated with the detection of more irreversible perfusion defects, but its effect on the extent of reversible perfusion abnormalities were not established. This means that beta-blocker treatment may induce a reduction in dipyridamole-phase perfusion similar to the reduction in resting perfusion in the areas of stenotic coronary arteries. The effect of beta-blocker treatment with metoprolol on the coronary flow rate of patients with severe CAD was assessed in another study in which the patients underwent dipyridamole NH3-PET imaging while randomly on and off beta-blocker medications [10]. In these patients, both resting and hyperemic blood flow in either stenotic or normal myocardial segments were decreased by beta-blocker treatment, in accordance with the finding of the present study [10]. There are two possible explanations for these results. Adrenergic blockade may blunt the adenosine effect and coronary vasodilatation during ischemia due to a direct interference with the adenosine receptor or adenosine concentration [10], resulting in less contrast between resting and post-dipyridamole images of the ischemic areas, possibly causing overestimation of the fixed defects. Another possible mechanism is that the systolic pressure tends to be decreased by beta-blockade during dipyridamole stress; the driving pressure might be reduced and thereby may reduce the myocardial perfusion in stenotic areas during dipyridamole- and rest-phase imaging [10]. Thus, beta-blocker withdrawal may represent more accurate estimation of the extent of fixed versus reversible defects.

On the other hand, the effect of continuing beta-blocker treatment on the heterogeneity of regional myocardial perfusion had no significant impact on the sensitivity of the DMPI in our study. As a result, regarding the acknowledged risks of discontinuation of anti-anginal medications before any diagnostic testing, on-medication imaging protocol may be considered adequate to reveal the presence of underlying CAD. Nevertheless, there are some inaccuracies in the estimation of myocardial defect size caused by beta-blocker treatment, e.g. overestimation in the size of fixed defects in our study as well as underestimation of dipyridamole-induced defect size in a previously reported study [43]. Therefore, for the accurate analysis of the size and extent of fixed versus reversible myocardial defects especially in a known case of previous insult or candidates for long-term risk stratification, withholding beta-blocker treatment may be considered on a case-by-case basis.

### Study limitations

Because of ethical considerations (unnecessary exposure to radiation, inherent risks to the stress phase of MPI-such as myocardial infarction, arrhythmia, etc.), we were unable to conduct a within-subject study to evaluate the effect of beta-blocker withdrawal on the sensitivity of DMPI in a single population of patients; however, the two groups in our study were comparable as far as the gender, age, type of radiotracer used for imaging and the extent of CAD were concerned. For future studies and to come into the conclusion that beta-blockers did not affect the sensitivity of the test using the same population with double measurement (one for continuation and the other for discontinuation) could be more justified. In this approach the potential confounder effects of baseline clinical characteristics of patients would be omitted.

Also the study was conducted in patients treated with four different beta-blocking agents. It would have been ideal to assess each beta-blocker disjointedly, but since the consumption of some kinds of beta-blockers such as carvedilol was very uncommon in our patients, a well-powered analysis of the data by considering each type of drug, individually, was unfeasible.

## Conclusion

No difference was noted between the sensitivity of DMPI with or without discontinuing of either selective or nonselective beta-blocking agents in our study. Thus, in view of the fact that medication discontinuation may cause the patients to face increased risk of heart events, discontinuing long-term beta-blocker treatment 3 days before DMPI is not suggested especially when only the diagnosis of CAD is the main concern. Beta-blocker withholding before MPI may be indicated on a case-by-case basis just in those patients referred for the accurate estimation of the size of fixed versus reversible defects.

## Competing interest

The authors declare that they have no competing interests.

## Authors’ contributions

DB was involved in all aspects of the study and the corresponding author. BF and DB designed and planed the study, deduced the data and revised the manuscript. AG did bibliography and drafted the article. AF and SA acquired data. FA, SI, JE interpreted data and revised the manuscript. AG and BF conducted the data analysis, interpreted data. MS and ME edited/revised the article. All authors read and approved the final manuscript.

## References

[B1] Zaman MU Hashmi I Fatima N Recent developments and future prospects of SPECT myocardial perfusion imaging Ann Nucl Med 2010 24 8 565 569 10.1007/s12149-010-0400-z 20652774

[B2] Cuocolo A Petretta M Acampa W De Falco T Gated SPECT myocardial perfusion imaging: the further improvements of an excellent tool Q J Nucl Med Mol Imaging 2010 54 2 129 144 20588210

[B3] Gholamrezanezhad A Mirpour S Esfehani AF Saghari M Mirpour K Beiki D Soheilifar M A correlative study comparing current different methods of calculating left ventricular ejection fraction Nucl Med Commun 2007 28 1 41 48 10.1097/01.mnm.0000237990.37325.74 17159548

[B4] Fallahi B Beiki D Gholamrezanezhad A Mahmoudian B Ansari Gilani K Eftekhari M Fard-Esfahani A Mohseni Z Saghari M Single 99mTc Sestamibi injection, double acquisition gated SPECT after stress and during low-dose dobutamine infusion: a new suggested protocol for evaluation of myocardial perfusion Int J Cardiovasc Imaging 2008 24 8 825 835 10.1007/s10554-008-9328-y 18563624

[B5] Baskot B Obradović S Gligić B Orozović V Ristić-Angelkov A Romanović R Jung R Ivanović V Bikicki M Pavlović M Adenosine stress protocols for myocardial perfusion imaging Vojnosanit Pregl 2008 65 1 47 50 10.2298/VSP0801047B 18368938

[B6] Bokhari S Ficaro EP McCallister BD Jr Adenosine stress protocols for myocardial perfusion imaging J Nucl Cardiol 2007 14 3 415 416 10.1016/j.nuclcard.2007.04.005 17556176

[B7] Gholamrezanezhad A Mirpour S Hajimohammadi H Pourmoslemi A Submaximal target heart rate and the detection of myocardial ischemia by stress myocardial perfusion imaging using the treadmill exercise Bruce protocol Anadolu Kardiyol Derg 2008 8 3 192 196 18524725

[B8] Patel RN Arteaga RB Mandawat MK Thornton JW Robinson VJ B Pharmacologic stress myocardial perfusion Imaging South Med J 2007 100 10 1006 1014 10.1097/SMJ.0b013e318153f9c6 17943047

[B9] Taillefer R Ahlberg AW Masood Y White CM Lamargese I Mather JF McGill CC Heller GV Acute beta-blockade reduces the extent and severity of myocardial perfusion defects with dipyridamole 99mTc-sestamibi SPECT imaging J Am Coll Cardiol 2003 42 8 1475 1483 10.1016/S0735-1097(03)01046-5 14563595

[B10] Bøttcher M Refsgaard J Madsen MM Randsbaek F Kaltoft A Bøtker HE Nielsen TT Effect of antianginal medication on resting myocardial perfusion and pharmacologically induced hyperemia J Nucl Cardiol 2003 10 4 345 352 10.1016/S1071-3581(03)00454-9 12900738

[B11] Shehata AR Gillam LD Mascitelli VA Herman SD Ahlberg AW White MP Chen C Waters DD Heller GV Impact of acute propranolol administration on dobutamine-induced myocardial ischemia as evaluated by myocardial perfusion imaging and echocardiography Am J Cardiol 1997 80 3 268 272 10.1016/S0002-9149(97)00344-5 9264417

[B12] Martin GJ Henkin RE Scanlon PJ Beta blockers and the sensitivity of the thallium treadmill test Chest 1987 92 3 486 487 10.1378/chest.92.3.486 2887403

[B13] Burns RJ Kruzyk GC Armitage DL Druck MN Effect of antianginal medications on the prognostic value of exercise thallium scintigraphy Can J Cardiol 1989 5 1 29 32 2563954

[B14] Bonaduce D Muto P Morgano G Pace L Ferrara N Salvatore M Condorelli M Effect of beta-blockade on thallium-201 dipyridamole myocardial scintigraphy Acta Cardiol 1984 39 6 399 408 6335336

[B15] Zoghbi GJ Dorfman TA Iskandrian AE The effects of medications on myocardial perfusion J Am Coll Cardiol 2008 52 6 401 416 10.1016/j.jacc.2008.04.035 18672159

[B16] Hurst RT Askew JW Lee R Resolution of myocardial bridge-related wall motion abnormality and associated myocardial perfusion defect with beta-blocker therapy J Invasive Cardiol 2005 17 12 E40 E42 16327037

[B17] Brandts B Dirkmann D Borchard R Wickenbrock I Van Bracht M Prull MW Trappe HJ Propranolol inhibits IK(Ado) by competitive A1-receptor interaction Z Kardiol 2004 93 7 533 539 15243764 10.1007/s00392-004-0094-0

[B18] Koepfli P Wyss CA Namdar M Klainguti M von Schulthess GK Lüscher TF Kaufmann PA Beta-adrenergic blockade and myocardial perfusion in coronary artery disease: differential effects in stenotic versus remote myocardial segments J Nucl Med 2004 45 10 1626 1631 15471825

[B19] Efird J Blocked randomization with randomly selected block sizes Int J Environ Res Public Health 2011 8 1 15 20 21318011 10.3390/ijerph8010015 PMC3037057

[B20] Dobson JG Jr Mechanism of adenosine inhibition of catecholamine-induced responses in heart Circ Res 1983 52 2 151 160 10.1161/01.RES.52.2.151 6297829

[B21] Wang T Mentzer RM Jr Van Wylen DG Interstitial adenosine with dipyridamole: effect of adenosine receptor blockade and adenosine deaminase Am J Physiol 1992 263 2 Pt 2 552 558 10.1152/ajpheart.1992.263.2.H552 1510152

[B22] Sopena M Viña J Calderón J Pallardó F Rodrigo F Canós J Cabo J In vitro study of inhibition of adenosine-deaminase by dipyridamole Rev Esp Fisiol 1970 26 4 303 306 5497997

[B23] Gresele P Arnout J Deckmyn H Vermylen J Mechanism of the antiplatelet action of dipyridamole in whole blood: modulation of adenosine concentration and activity Thromb Haemost 1986 55 1 12 18 3704998

[B24] Meisel P Meisel M Grisk A Some properties of adenosine deaminase from vascular smooth muscle and its inhibition by various vasodilators Cor Vasa 1976 18 1 56 65 1261276

[B25] Gould KL Lipscomb K Calvert C Compensatory changes of the distal coronary vascular bed during progressive coronary constriction Circulation 1975 51 6 1085 1094 10.1161/01.CIR.51.6.1085 1132098

[B26] Gould KL Lipscomb K Effects of coronary stenoses on coronary flow reserve and resistance Am J Cardiol 1974 34 1 48 55 10.1016/0002-9149(74)90092-7 4835753

[B27] Flameng W Wüsten B Schaper W On the distribution of myocardial flow Part II: Effects of arterial stenosis and vasodilation. Basic Res Cardiol 1974 69 4 435 446 10.1007/BF01905946 4441338

[B28] Wüsten B Flameng W Schaper W The distribution of myocardial flow Part I: Effects of experimental coronary occlusion. Basic Res Cardiol 1974 69 4 422 434 10.1007/BF01905945 4441337

[B29] Flameng W Schaper W Wüsten B Myocardial blood flow distribution in experimental coronary occlusion without myocardial infarct Verh Dtsch Ges Kreislaufforsch 1973 39 199 202 10.1007/978-3-642-85288-6_37 4785927

[B30] Becker LC Conditions for vasodilator-induced coronary steal in experimental myocardial ischemia Circulation 1978 57 6 1103 1110 10.1161/01.CIR.57.6.1103 416923

[B31] Johnson JA Liggett SB Cardiovascular pharmacogenomics of adrenergic receptor signaling: clinical implications and future directions Clin Pharmacol Ther 2011 89 3 366 378 10.1038/clpt.2010.315 21289619 PMC3110683

[B32] Wallukat G The beta-adrenergic receptors Herz 2002 27 7 683 690 10.1007/s00059-002-2434-z 12439640

[B33] Herrmann SC Feigl EO Adrenergic blockade blunts adenosine concentration and coronary vasodilation during hypoxia Circ Res 1992 70 6 1203 1216 10.1161/01.RES.70.6.1203 1315635

[B34] Ferrara N Coltorti F Leosco D Sederino S Abete P Caccese P Landing P Longobardi G Verde R Rengo F Protective effect of beta-blockade on dipyridamole-induced myocardial ischaemia Role of heart rate. Eur Heart J 1995 16 7 903 908 10.1093/oxfordjournals.eurheartj.a061023 7498204

[B35] Hockings B Saltissi S Croft DN Webb-Peploe MM Effect of beta adrenergic blockade on thallium-201 myocardial perfusion imaging Br Heart J 1983 49 1 83 89 10.1136/hrt.49.1.83 6821615 PMC485214

[B36] Sharir T Rabinowitz B Livschitz S Moalem I Baron J Kaplinsky E Chouraqui P Underestimation of extent and severity of coronary artery disease by dipyridamole stress thallium-201 single-photon emission computed tomographic myocardial perfusion imaging in patients taking antianginal drugs J Am Coll Cardiol 1998 31 7 1540 1546 10.1016/S0735-1097(98)00142-9 9626832

[B37] Kubota I Ikeda K Igarashi H Kawashima S Yamaki M Yasumura S Tsuiki K Yasui S Inhibition of dipyridamole-induced myocardial ischemia by diltiazem in patients with coronary artery disease J Cardiovasc Pharmacol 1987 9 3 363 367 10.1097/00005344-198703000-00013 2437403

[B38] Lattanzi F Picano E Bolognese L Piccinino C Sarasso G Orlandini A L’Abbate A Inhibition of dipyridamole-induced ischemia by antianginal therapy in humans. Correlation with exercise electrocardiography Circulation 1991 83 4 1256 1262 10.1161/01.CIR.83.4.1256 1672841

[B39] Reyes E Stirrup J Roughton M D’Souza S Underwood SR Anagnostopoulos C Attenuation of adenosine-induced myocardial perfusion heterogeneity by atenolol and other cardioselective beta-adrenoceptor blockers: a crossover myocardial perfusion imaging study J Nucl Med 2010 51 7 1036 1043 10.2967/jnumed.109.073411 20554740

[B40] Bridges AB Kennedy N McNeill GP Cook B Pringle TH The effect of atenolol on dipyridamole 201Tl myocardial perfusion tomography in patients with coronary artery disease Nucl Med Commun 1992 13 1 41 46 10.1097/00006231-199201000-00007 1594169

[B41] Lakkireddy D Aronow WS Bateman T McGhee I Nair C Khan IA Does beta blocker therapy affect the diagnostic accuracy of adenosine single-photon-emission computed tomographic myocardial perfusion imaging? Am J Ther 2008 15 1 19 23 10.1097/MJT.0b013e31804c71a7 18223349

[B42] Yoon AJ Melduni RM Duncan SA Ostfeld RJ Travin MI The effect of beta-blockers on the diagnostic accuracy of vasodilator pharmacologic SPECT myocardial perfusion imaging J Nucl Cardiol 2009 16 3 358 367 10.1007/s12350-009-9066-0 19266249

[B43] Gerson MC Reduction in dipyridamole-induced single-photon emission computed tomography myocardial defect size by beta-blockers: time to re-examine the patient preparation protocol for pharmacologic stress testing J Am Coll Cardiol 2003 42 8 1484 1486 10.1016/S0735-1097(03)01047-7 14563596

